# Aviation Contrail Cirrus and Radiative Forcing Over Europe During 6 Months of COVID‐19

**DOI:** 10.1029/2021GL092771

**Published:** 2021-04-28

**Authors:** U. Schumann, L. Bugliaro, A. Dörnbrack, R. Baumann, C. Voigt

**Affiliations:** ^1^ Institute of Atmospheric Physics Deutsches Zentrum für Luft‐ und Raumfahrt Oberpfaffenhofen Germany; ^2^ Johannes Gutenberg‐University Mainz Germany

**Keywords:** aviation, cirrus, contrail, COVID‐19, radiative forcing, satellite

## Abstract

The COVID‐19 pandemic led to a 72% reduction of air traffic over Europe in March–August 2020 compared to 2019. Modeled contrail cover declined similarly, and computed mean instantaneous radiative contrail forcing dropped regionally by up to 0.7 W m^−2^. Here, model predictions of cirrus optical thickness and the top‐of‐atmosphere outgoing longwave and reflected shortwave irradiances are tested by comparison to Meteosat‐SEVIRI‐derived data. The agreement between observations and modeled data is slightly better when modeled contrail cirrus contributions are included. The spatial distributions and diurnal cycles of the differences in these data between 2019 and 2020 are partially caused by differences in atmospheric and surface conditions, particularly for solar radiation in the spring of 2020. Aviation signals become discernible in the observed differences of these data between 2019 and 2020 when subtracting numerical weather prediction model results that approximate the atmosphere and surface conditions without contrails.

## Key Points

- Air traffic reduction during the COVID‐19 pandemic is used for testing aviation contrail cirrus and related radiative forcing modeling
- Cirrus optical depth and top‐of‐atmosphere irradiances from satellite observations and model results show reasonable correlations
- Aviation contrail signatures are identifiable in spite of strongly different atmosphere and surface conditions in 2019 and 2020

## 1 Introduction

Contrails induced by water vapor and soot emissions from aircraft flying in cold and humid air masses are expected to have caused more than half of the about 0.1 W m^−2^ global effective radiative forcing (RF) by aviation in recent years (Lee et al., 2021). Contrails impact climate by positive longwave RF because they reduce top‐of‐atmosphere (TOA) outgoing longwave radiation (OLR), leading to a warming of the Earth‐atmosphere system. In addition, contrails cause negative shortwave RF by reflecting solar radiation (RSR) inducing system cooling (Meerkötter et al., 1999; Minnis et al., 1999; Myhre et al., 2009). Most studies conclude that the net RF (sum of TOA instantaneous longwave and shortwave contrail RF) is positive (Boucher et al., 2013; Burkhardt & Kärcher, 2011; Fuglestvedt et al., 2010; Lee et al., 2021; Penner et al., 1999; Sausen et al., 2005; Schumann et al., 2015). Still, contrails and aircraft emissions impact the atmosphere in a complex manner and not all aspects are well understood (Brasseur et al., 2016; Kärcher, 2018; Lee et al., 2021; Schumann & Heymsfield, 2017). Uncertainty arises, e.g., due to possible contributions from cloud changes induced by indirect effects of aerosol from aircraft exhaust, possibly including soot released from sublimating contrail ice crystals (Lee et al., 2021; Penner et al., 2018; Urbanek et al., 2018). Reducing warming contrails would have an immediate impact on aviation RF, while reducing CO_2_ emissions reduces the forcing only slowly (Penner et al., 1999). Therefore, minimizing contrail RF by route changes may be an option for a more sustainable air transport (Schumann et al., 2011). Such mitigation options require reliable estimates of the contrail RF and accurate predictions of their regional and temporal distributions (Teoh et al., 2020).

The contrail‐induced RF is difficult to derive from observations. Early studies adjusted modeled cover and optical properties of contrail cirrus to match observations and then estimated RF with radiative transfer models (Meyer et al., 2002; Minnis et al., 1999; Stuber et al., 2006). Others estimated RF from observed multiyear trends in contrails, or cirrus cloudiness, with a factor estimating the RF from the added cirrus (Boucher, 1999; Eleftheratos et al., 2007; Minnis et al., 2004; Stordal et al., 2005; Stubenrauch & Schumann, 2005; Zerefos et al., 2003). The forcing can be derived directly from differences in irradiances from satellite observations in contrail regions relative to irradiances in neighboring contrail‐free regions (Spangenberg et al., 2013; Vázquez‐Navarro et al., 2015). This approach requires clearly identifiable and well separated contrail lines, outside dense traffic regions (Bedka et al., 2013; Mannstein et al., 2010). A second direct approach, applicable to strong contrail cirrus outbreaks, derives RF from differences between observed irradiances in contrail regions with irradiances computed by a model without contrails (Haywood et al., 2009). A third approach derives aviation‐induced cirrus and irradiance changes from long‐term cirrus and irradiance observations correlated with systematic traffic patterns such as the diurnal traffic double‐wave over the North Atlantic (Graf et al., 2012). This worked well for longwave irradiances (Schumann & Graf, 2013). However, the shortwave component could not be derived this way because its diurnal cycle is dominated by the solar cycle and only weakly sensitive to the diurnal traffic cycle.

Since March 2020, various measures to curb the COVID‐19 pandemic led to a worldwide reduction of air traffic (ICAO, 2021; Schumann et al., 2021). This can be viewed as an unintended opportunity for testing model predictions on the RF by aviation. We therefore started to model aviation‐induced cirrus and irradiance changes and to collect comparable observation data (Voigt et al., 2020).

This paper investigates whether the traffic changes have measurable impact on cirrus coverage and optical thickness (OT) and TOA atmosphere irradiances. The investigation domain (20°W–20°E, 35°N–60°N) includes parts of Europe and the North Atlantic, with dense air traffic, for the time periods March to August in 2019 and in 2020. The observations are derived from the Spinning Enhanced Visible and Infrared Radiometer (SEVIRI) aboard the Meteosat Second Generation (MSG) satellite (Schmetz et al., 2002). The contrails are simulated with the Contrail Cirrus Prediction (CoCiP) model (Schumann, 2012). The model uses deterministic, high‐resolution operational analyses, and short‐term forecasts of the European Centre for Medium‐Range Weather Forecasts (ECMWF) Integrated Forecast System (IFS) (Bauer et al., 2015).

We compare differences of observed and modeled OT, OLR, and RSR between comparable seasons in 2019 and 2020 over Europe, i.e., between periods with high and low air traffic. We search for comparable signatures in the spatial distribution and in the diurnal cycle of mean values in dense traffic areas. We expect that the observed changes are partly due to different atmospheric and surface conditions (“weather”) in the compared time periods (van Heerwaarden et al., 2021) and partly due to different air traffic, besides other anthropogenic and natural emissions (Guevara et al., 2021; Le Quéré et al., 2020). The weather conditions are represented by the ECMWF forecasts, assuming that the IFS simulates the atmosphere without aviation effects. Changes due to different air traffic are assumed to be dominated by contrail effects simulated with CoCiP.

## 2 Model and Observation Data

The contrail model uses traffic data from the European Organization for the Safety of Air Navigation (EUROCONTROL) and the UK air navigation service provider NATS (Schumann et al., 2021). Fuel consumption, overall propulsion efficiency, and soot emissions are estimated using the Base of Aircraft Data (BADA3) from EUROCONTROL (Nuic et al., 2010), an open‐access performance model PS (Poll & Schumann, 2021), and a fractal aggregate soot model (Teoh et al., 2019). The air traffic data, the forecast data from the ECMWF IFS, and the CoCiP model results, including contrail‐contrail overlap effects (Sanz‐Morère et al., 2021), are described and discussed in a recent study (Schumann et al., 2021).

Below, we summarize the observation methods used to derive ice cloud OT and OLR and RSR irradiances. The observation methods provide contrail cirrus and irradiance properties, without reference to line‐shaped contrail patterns, as appropriate for dense traffic cases.

Ice cloud and contrail properties are derived hourly from thermal observations of the sensor SEVIRI aboard the geostationary MSG satellite (Meteosat‐11) operating at 0°E, with data provided by EUMETSAT (Schmetz et al., 2002). MSG‐SEVIRI is a 12‐channel radiometer scanning the Earth disk with 4.2‐km mean spatial surface resolution in the investigation domain. The occurrence and OT of ice clouds including contrails is derived using the Cirrus Properties from SEVIRI (CiPS) algorithm (Strandgren et al., 2017a, 2017b). The method uses a set of four neural networks trained with cloud products from the CALIOP lidar aboard CALIPSO (Winker et al., 2009). The estimated mean absolute errors against CALIOP are smaller than 50%.

The irradiance, i.e., the amount of radiant flux impinging on a unit surface area, of both the RSR and the OLR are derived from SEVIRI observations using the Rapid Retrieval of Upwelling irradiances from MSG‐SEVIRI (RRUMS) algorithm (Vázquez‐Navarro et al., 2013). The shortwave irradiances are computed from the three purely solar channels of SEVIRI while the longwave part is derived from its 7 thermal channels. The solar retrieval uses a neural network, the thermal algorithm uses a linear combination of brightness temperatures (Vázquez‐Navarro et al., 2013). Both exploit an extensive set of radiative transfer simulations performed with the libRadtran radiative transfer model (Emde et al., 2016; Vázquez‐Navarro et al., 2013). Compared to the Clouds and the Earth’s Radiant Energy System (CERES) (Wielicki et al., 1996) and the Geostationary Earth Radiation Budget (GERB) instruments (Harries et al., 2005), RRUMS combines high temporal and high spatial resolution as required for the study of short lived and small scale features like contrails (Schumann & Graf, 2013; Vázquez‐Navarro et al., 2015). The mean bias for a single scene is estimated to be smaller than 5 W m^−2^ for OLR and of order 10 W m^−2^ for RSR with root‐mean square (rms) values of order 10 W m^−2^ and 30 W m^−2^ for OLR and RSR respectively based on various validation results (Vázquez‐Navarro et al., 2013). The RSR is determined for solar zenith angles (SZA) below 78.4° (cos(SZA) > 0.2) and set to zero otherwise. The same cut‐off is applied to model data. As a consequence, the computed shortwave RF is slightly smaller than for the full solar cycle.

## 3 Results

### 3.1 Mean Distributions

Air traffic over Europe is maximum along the traffic routes between UK and the Balkan countries, and between Scandinavia and the Iberian Peninsula. The mean traffic distances and fuel consumption declined by 72% in 2020 on average over the 6 months compared to the same month in 2019. The reductions reached 91% in April 2020 (Schumann et al., 2021).

**Figure 1:**
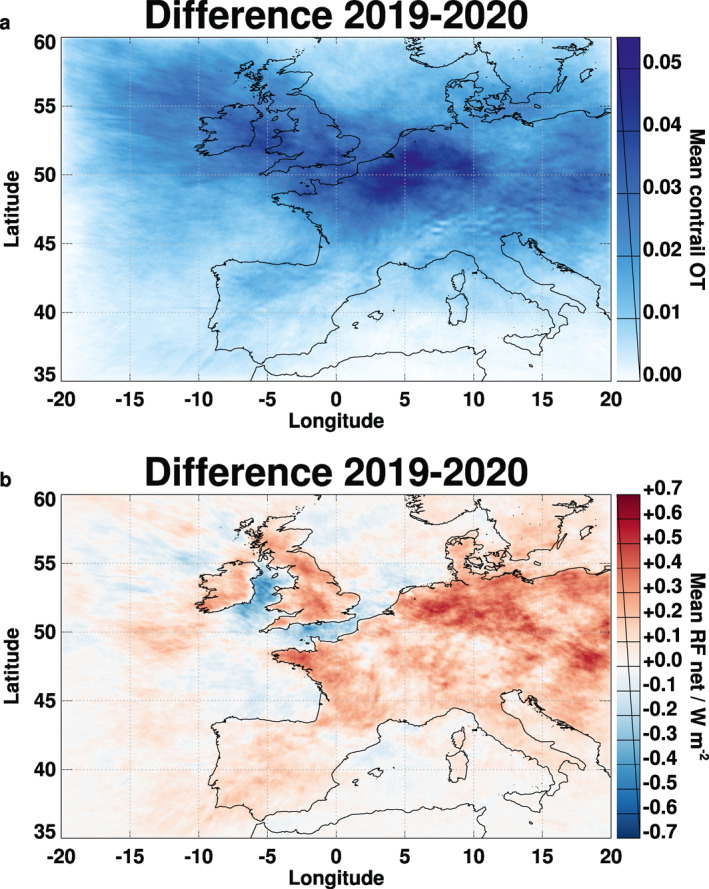
Mean differences of modeled optical depth (top) and net RF (bottom) of contrails between March–August 2019 and March–August 2020. RF, radiative forcing.

Most contrails occur near flight level (FL) 350 (10.7 km pressure altitude) which is often cold and humid enough for the formation of persistent contrails. The spatial distributions of the computed differences in mean OT of contrail cirrus and of the local net RF due to contrails between 2019 and 2020 are shown in Figure 1. The mean area‐coverage of contrails with an OT larger than 0.1 decreased from 4.6% in 2019 to 1.4% in 2020. The 2019–2020 OT differences are mostly positive and largest along an about 600‐km wide and 1,500‐km long band between the Atlantic northwest of Ireland and the Balkan countries. Apparently, this is a zone with strong traffic reductions and suitable conditions for contrail formation at upper‐tropospheric FLs. The corresponding net RF reductions are mostly positive. Negative differences occur over the ocean northwest of Ireland, the Irish Sea, and the English Channel because of regionally dominating shortwave RF over low solar albedo surfaces.

The 6‐month and domain‐mean air traffic density, fuel consumption, contrail OT, longwave RF, and shortwave RF are 0.11 kg km^−2^ h^−1^, 0.40 kg km^−2^ h^−1^, 0.018, 0.90 W m^−2^, and −0.76 W m^−2^ in 2019, respectively, with about (72 ± 2)% smaller values in 2020. The 6‐month mean 2019–2020 differences reach extreme values of 0.054, 2.2 W m^−2^ and −2.1 W m^−2^ for OT, longwave RF, and shortwave RF. Due to various nonlinearities, net RF decreased by 54%. These values apply for the reference case; parameter studies show an uncertainty of order 50% (Schumann et al., 2021).

**Figure 2:**
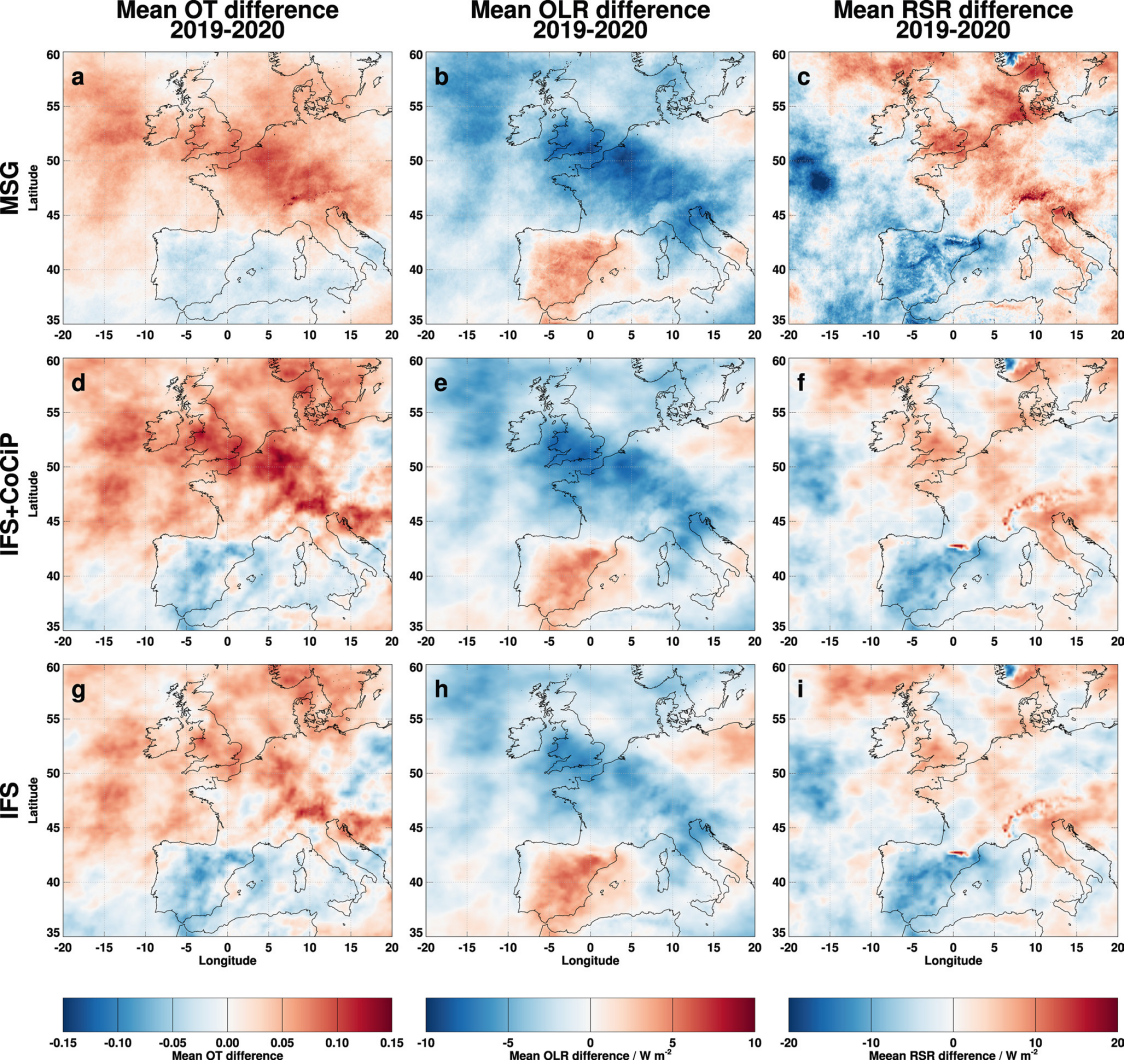
Mean differences of (a–c) observed, (d–f) modeled with contrails (IFS + CoCiP), and (g–i) modeled without contrails (IFS) ice cloud optical depth (OT), outgoing longwave irradiance (OLR), and reflected shortwave irradiance (RSR) in March‐August between 2019 and 2020. IFS, Integrated Forecast System; CoCiP, Contrail Cirrus Prediction.

Figure 2 compares spatial distributions of observation and model results of OT, OLR and RSR in terms of mean differences between 2019 and 2020. Areas with enhanced cirrus cloudiness and negative longwave and positive shortwave irradiances in 2019 compared to 2020 appear along a band from the North Atlantic northwest of Ireland to the Balkan countries. Differences with opposite sign occur also, e.g., over Spain. Regional contrail signatures (higher OT, lower OLR, and higher RSR) can be identified between UK and the Balkan countries after subtracting the last row (IFS) from the first (MSG) and second (IFS + CoCiP) rows (supporting information S1). Here, we assume that model and observation errors do not dominate the observed signatures. The mean observed and modeled spatial difference distributions show similarities which are quantified in terms of the Pearson correlation coefficient (*r* ^2^), a normalized mean bias (NMB), and the root‐mean‐square error (RMSE) (defined in supporting information S2), see Table 1. The correlations are highest for OLR, followed by RSR and by OT and higher in spring (MAM) than in the full half‐year period. In most cases, the correlations increase when including the contrail contributions (not for RSR in spring). The NMB and RMSE values decrease for OLR and OT, but not for RSR. So, the model‐observation agreement increases for OT and OLR when the contrail model is included.

**Table 1:** Comparisons Between Measured and Modeled Mean Differences 2020–2019 for 6‐Months or 3‐Months Mean Values (March‐August and March‐May), Either Without Contrail Model (IFS Only) or With Contrail Model Results

Period	Parameter	Without contrail model			With contrail model			RMSE Unit
		*r* ^2^ (%)	NMB (%)	RMSE	*r* ^2^ (%)	NMB (%)	RMSE	
MAR‐AUG	OT	77.7	−21.4	0.024	85.4	9.8	0.022	1
	OLR	93.1	29.6	1.46	95.2	13.5	0.98	W m^−2^
	RSR	82.8	5.5	3.57	82.9	12.9	3.60	W m^−2^
MAR‐MAY	OT	88.7	−30.8	0.040	91.1	−12.8	0.034	1
	OLR	97.7	9.1	1.81	97.9	1.3	1.55	W m^−2^
	RSR	92.7	4.2	5.31	92.4	7.7	5.42	W m^−2^

*Note*. Often used acronyms (for a complete list, see Supplement): CiPS, Cirrus Properties from SEVIRI; CoCiP, Contrail Cirrus Prediction; FL, flight level; IFS, Integrated Forecasting System of the European Center for Medium‐Range Weather Forecasts; LW, longwave; OLR, outgoing longwave radiation; OT, optical thickness; RF, radiative forcing; RRUMS, Rapid Retrieval of Upwelling irradiances from MSG‐SEVIRI; RSR, reflected shortwave radiation; SEVIRI, Spinning Enhanced Visible and Infrared Radiometer; SW, shortwave; TOA, top‐of‐atmosphere.

### 3.2 Mean Diurnal Cycles

The weekly cycles of traffic and contrail properties on average over the investigation domain in the two half‐year periods in 2019 and 2020 (supporting information S1) indicate large changes for each day and a weak modulation during the week. Traffic is maximum at noon, with pronounced minima at midnight. Since the contrail lifetimes are finite (∼2‐h mean age), the contrail cover follows the traffic with a corresponding delay, reaching maximum in early afternoon (Graf et al., 2012). The longwave RF signal follows mainly the contrail cover which indicates a minor diurnal variability of other drivers like contrail temperature. The shortwave RF is modulated mainly by the diurnal cycle of the solar zenith angle (Meerkötter et al., 1999; Myhre & Stordal, 2001). As a consequence, net RF is positive during night, but negative in the afternoon. Details depend amongst others on the ice particle scattering properties and their modeling (Markowicz & Witek, 2011; Schumann et al., 2012). The weekly cycle shows slightly stronger traffic between Friday and Sunday than in the rest of the week. The contrail properties should show similar weekly modulations with maxima at end of the week. The correlations are not perfect, as expected for only 26‐weeks mean values.

**Figure 3:**
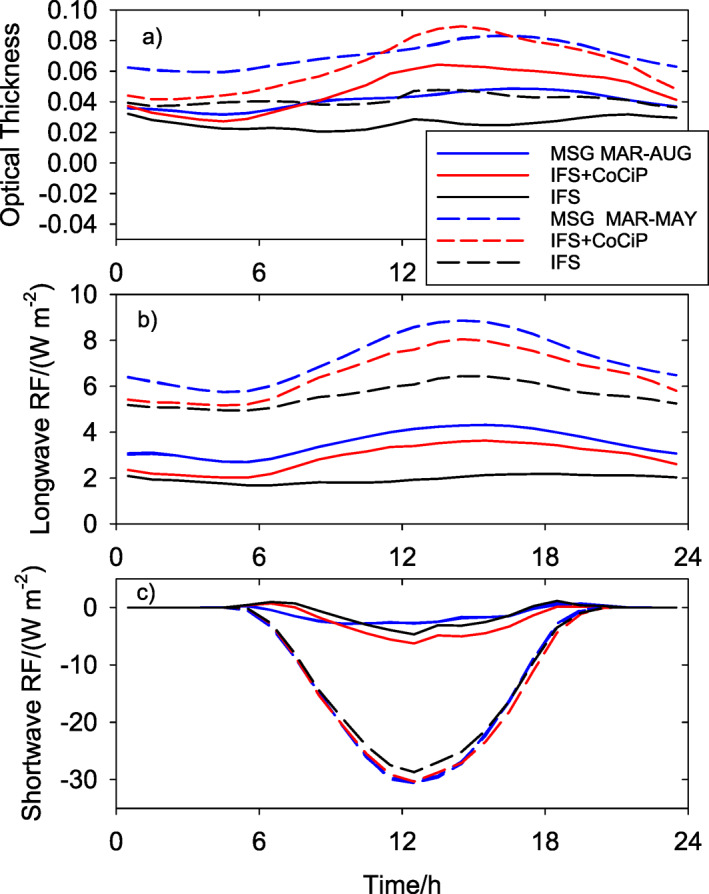
Mean diurnal cycles of area‐averaged 2019–2020 differences of (a) ice cloud optical thickness, (b) longwave RF (negative OLR difference), and (c) shortwave RF (negative RSR difference) for March‐August (full) and March‐May (dashed). Blue: Observations from MSG, red: combined CoCip + IFS model result, black: pure IFS model result. The area‐average is taken over the European domain weighted with the sum of optical depth values of contrails in 2019 and 2020. RF, radiative forcing; OLR, outgoing longwave irradiance; IFS, Integrated Forecast System; MSG, Meteosat Second Generation.

Figure 3 shows the mean diurnal cycle of area‐averaged differences of OT, longwave RF, and shortwave RF. The interannual differences in longwave and shortwave RF equal the negative differences in OLR and RSR. The area‐average is computed with weights constant in time and spatially proportional to the sum of the computed contrail OT in the two years, similar to Figure 1.

For dominating aviation effects, with time scales of hours, we would expect temporally variable positive OT differences, positive longwave RF, and a more negative shortwave RF in late afternoon than in the morning for stronger traffic in 2019 than in 2020. This is what is shown by the model results in Figure 3. The diurnal cycles show nonzero mean differences between 2019 and 2020 plus temporally variable values. The mean values have large‐scale causes, constant over a day, and cannot be explained by the shorter‐lived contrails. For shortwave radiation, a constant increase in surface albedo would cause an increase in RSR (reduction in shortwave RF) modulated by the incoming solar irradiance. The mean difference 2019–2020 is positive for OT and for longwave RF; and the negative differences in shortwave RF are larger in MAM than for the full 6‐month period. Hence, large parts of the differences are caused by different atmospheric and surface conditions, and modulated by the aviation effects.

The temporally variable 2019–2020 differences of MSG‐SEVIRI observations correlate with the fluctuations in the model results including contrail contributions to some degree, not perfectly, but better than with the corresponding differences of IFS contributions only. The correlations are highest for longwave RF. The longwave RF is positive and larger during afternoon than after midnight, in phase with contrail cover. For OT, it appears as if the combined model computes values that are too large, or other effects (e.g., soot changes of ambient cirrus) may also contribute changes in OT. For shortwave RF, the diurnal cycles are very different between spring and summer. Spring 2019 had about 30 W m^−2^ less shortwave RF at noon than 2020, far more than explainable by aviation effects. Apparently, 2020 had a lower Earth albedo, i.e., less cloudiness and darker surfaces. The IFS data show similar reductions. So, much of this shortwave RF change is due to the different weather conditions covered by the IFS.

## 4 Discussion

The 2019–2020 differences in the modeled contrail cirrus OT (Figure 1) follow the corresponding changes in air traffic and fuel consumption (supporting information S1) but are far smoother in space because of contrail dispersion during the up to 16 h contrail lifetime. The OT differences are largest over Central Europe, not only because of maximum traffic changes but also because of suitable atmospheric conditions in that region. The net RF differences shown in Figure 1 resemble different responses in the longwave and shortwave parts, depending on solar irradiance, surface, ambient atmosphere, and contrail properties, and therefore contrail properties are not always proportional to the OT differences (Meerkötter et al., 1999; Schumann et al., 2012). The net RF changes with maximum of 0.7 W m^−2^ would have been about 30–50% larger in a model without contrail‐contrail overlap effects (Sanz‐Morère et al., 2021; Schumann et al., 2021).

The observation and model results (Figure 2) show large differences in OT, OLR, and RSR over Europe between 2019 and 2020. The signs of the differences are mostly as expected for higher traffic activity in 2019 compared to 2020 (increase in OT, decrease in OLR or increase in instantaneous longwave RF, increase in RSR or decrease of instantaneous shortwave RF), but the changes are too large to be caused by aviation alone. Figure 2 also reveals regional differences with signs opposite to the expected aviation effects (e.g., over Spain), presumably from different weather conditions. Moreover, other effects like surface emissions and related changes in low‐level clouds and interannual differences in model or observation biases might contribute to the differences as well.

The correlations between observations and model results (Table 1) are all positive, between 78% and 98%, with normalized biases in the 20% range. This is within the range of expected observation and model errors and reflects the quality of the observations and the predictive skill of the IFS model. The correlations increase by a few percent and the biases decrease to about 10% when the CoCiP model results are included, at least for OT and OLR, as they should when aviation contrail effects are important. The agreement is best for OLR, which is sensitive to cirrus changes, with the lower troposphere humidity shielding part of the surface radiation, whereas RSR is more sensitive to surface albedo changes (Gristey et al., 2018; Stephens, 2005). Part of the RSR changes may be caused by low‐level clouds for reduced surface emissions. Hence, the RSR changes are dominated by nonaviation effects.

The modeled OT appears to be about 20% too large compared to the observations. The model may overestimate OT by overestimating solar extinction, which is modeled as a function of the IFS temperature and ice water content using an empirical parameterization with about 30% uncertainty (Heymsfield et al., 2014). The CiPS cirrus OT was calibrated with space lidar observations and saturates at OT values of 3–5 (Strandgren et al., 2017b). Therefore, these deviations are within the expected range.

The interannual differences in atmospheric states can be derived from the IFS data. The IFS represents the atmosphere without contrails, since it does not simulate contrails, and the only cloud‐affected radiance observations that are assimilated are in the microwaves at frequencies insensitive to thin ice clouds (Geer et al., 2018; Greenwald & Christopher, 2002; Hogan & Bozzo, 2018). The IFS data (supporting information S5 and S6) reveal differences in ambient conditions between 2020 and 2019. These are larger in spring (MAM) than in summer (JJA), different in the northern and southern parts of the domain, and stronger in the lower troposphere than at flight levels. As noted by van Heerwaarden et al. (2021), spring 2020 was exceptionally sunny, warm, and dry in the northern parts of the European domain, with a “blue sky,” inducing 18 W m^−2^ more surface global solar irradiance on average. OLR and the planetary albedo also differed strongly, in particular in spring in the northern part of the domain (by 7 W m^−2^ and by 2%). Summer 2020 was more similar to 2019.

The direct impact of different atmospheric states on contrail formation is smaller: At FL 350, temperature is about 1 K higher in 2020 than in 2019, with slightly higher absolute, but lower relative humidity. The potential contrail cover (i.e., the area fraction with temperature below the contrail formation threshold and humidity above ice saturation (Sausen et al., 1998; Schumann, 1996)) decreases from 16% in 2019 to 15% in 2020 (supporting information S5 and S7). This is also supported by simulations of RF for the 2019 meteorology with air traffic from 2020 (Schumann et al., 2021) yielding very similar values to the simulation for 2020 (meteorology and air traffic).

Besides contrails, other aviation effects might have contributed to the observed differences between 2019 and 2020. Greenhouse effects from long‐lived emitted CO_2_ and NO_*x*_‐induced changes of CH_4_ take years to become important (Lee et al., 2010) and are too small to be noticeable after a few months. Ozone changes induced by NO_*x*_ take days to weeks to reach a new equilibrium after emission (Brasseur et al., 2016) and they cannot explain differences at the hourly time scales as observed in the diurnal cycle (Figure 3). Emission changes outside Europe could have contributed to the mean values in this figure, but the fact that the maximum changes occurred quickly after the emission reduction started in spring 2020 indicates that these emissions are less important. Soot emissions and other aviation‐induced aerosols could change cirrus clouds regionally, but this process is slower than contrail formation (Zhou & Penner, 2014) and, hence, has less impact on the variable part of the diurnal cycle. The diurnal cycles (Figure 3) do not suggest a cooling from such aerosol because the mean longwave RF differences are positive and the mean shortwave RF differences are insignificant in the 6‐month period.

## 5 Conclusions

Contrail and weather‐induced cirrus and irradiance changes during the COVID‐19 pandemic are derived from SEVIRI observations and model results. Spring 2020 was far sunnier than spring 2019 contributing to a “blue sky” over large parts of Europe, with summer months more similar in the two years. The changes at FL were smaller, 1 K warmer on average, with slightly less potential contrail cover in 2020.

The contrail‐induced cirrus and irradiance modulations are smaller than the weather‐induced 2019–2020 differences, but aviation signatures are detectable. The modeled OT may overestimate the observed cirrus OT, possibly due to an overestimate of cirrus in the models and potential observation underestimates. Different from RSR, which is strongly sensitive to surface albedo and low‐level clouds, OLR is less sensitive to low‐level changes because of partial infrared shielding by water vapor. The overall model vs. observation agreement, with correlations of order 90%, support the validity of the models. The IFS is likely to be responsible for most of this correspondence, but the correlations increase (except for RSR) and the normalized biases drop below 15% when the contrail model is included.

The mean diurnal cycle reveals mean differences in OT, OLR, and RSR between 2019 and 2020 from contributions on time scales beyond days and overlaid on that variations during the day. The variable parts of the observations correlate best with modeled values for OLR, consistent with the expected negative OLR difference from increased air traffic longwave RF. The RSR variability is too large to conclude definitely on aviation effects. None of the results indicate a strong misrepresentation of contrail effects in the models. However, because of the different atmospheric and surface conditions in 2019 and 2020, a quantitative assessment of the contrail model validity is beyond the information content of the data.

Since worldwide traffic activity has stayed at low levels after August 2020, with no end currently in sight (ICAO, 2021), these observations and comparisons may be extended into the future. Past years with strong traffic increases should be studied similarly. Further model components that simulate the influence of other emissions would allow an extension of the explanations of the observations.

## Supporting information

Supporting Information S1

## Data Availability

The model and observation results are available from http://doi.org/10.5281/zenodo.4481680.

## References

[grl62242-bib-0001] Bauer, P. , Thorpe, A. , & Brunet, G. (2015). The quiet revolution of numerical weather prediction. Nature, 525(7567), 47–55. 10.1038/nature14956 26333465

[grl62242-bib-0002] Bedka, S. T. , Minnis, P. , Duda, D. P. , Chee, T. L. , & Palikonda, R. (2013). Properties of linear contrails in the Northern Hemisphere derived from 2006 Aqua MODIS observations. Geophysical Research Letters, 40, 772–777. 10.1029/2012GL054363

[grl62242-bib-0003] Boucher, O. (1999). Air traffic may increase cirrus cloudiness. Nature, 397, 30–31. 10.1038/16169

[grl62242-bib-0004] Boucher, O. , Randall, D. , Artaxo, P. , Bretherton, C. , Feingold, G. , Forster, P. , et al. (2013). Clouds and aerosols. In T. F. Stocker , D. Qin , G.‐K. Plattner , M. Tignor , S. K. Allen , J. Boschung , et al. (Eds.), Climate change 2013: The physical science basis (pp. 571–657). Cambridge, UK and New York, NY: Cambridge University Press.

[grl62242-bib-0005] Brasseur, G. P. , Gupta, M. , Anderson, B. E. , Balasubramanian, S. , Barrett, S. , Duda, D. , et al. (2016). Impact of aviation on climate: FAA's Aviation Climate Change Research Initiative (ACCRI) phase II. Bulletin of the American Meteorological Society, 97(4), 561–583. 10.1175/BAMS-D-13-00089.1

[grl62242-bib-0006] Burkhardt, U. , & Kärcher, B. (2011). Global radiative forcing from contrail cirrus. Nature Climate Change, 1, 54–58. 10.1038/NCLIMATE1068

[grl62242-bib-0007] Eleftheratos, K. , Zerefos, C. S. , Zanis, P. , Balis, D. S. , Tselioudis, G. , Gierens, K. , & Sausen, R. (2007). A study on natural and manmade global interannual fluctuations of cirrus cloud cover for the period 1984–2004. Atmospheric Chemistry and Physics, 7, 2631–2642. 10.5194/acp-7-2631-2007

[grl62242-bib-0008] Emde, C. , Buras‐Schnell, R. , Kylling, A. , Mayer, B. , Gasteiger, J. , Hamann, U. , et al. (2016). The libRadtran software package for radiative transfer calculations (version 2.0.1). Geoscientific Model Development, 9, 1647–1672. 10.5194/gmd-9-1647-2016

[grl62242-bib-0009] Fuglestvedt, J. S. , Shine, K. P. , Berntsen, T. , Cook, J. , Lee, D. S. , Stenke, A. , et al. (2010). Transport impacts on atmosphere and climate: Metrics. Atmospheric Environment, 44(37), 4648–4677. 10.1016/j.atmosenv.2009.04.044 PMC7110594 32288556

[grl62242-bib-0010] Geer, A. J. , Lonitz, K. , Weston, P. , Kazumori, M. , Okamoto, K. , Zhu, Y. , et al. (2018). All‐sky satellite data assimilation at operational weather forecasting centres. Quarterly Journal of the Royal Meteorological Society, 144, 1191–1217. 10.1002/qj.3202

[grl62242-bib-0011] Graf, K. , Schumann, U. , Mannstein, H. , & Mayer, B. (2012). Aviation induced diurnal North Atlantic cirrus cover cycle. Geophysical Research Letters, 39, L16804. 10.1029/2012GL052590

[grl62242-bib-0012] Greenwald, T. J. , & Christopher, S. A. (2002). Effect of cold clouds on satellite measurements near 183 GHz. Journal of Geophysical Research, 107(D13), 4170. 10.1029/2000JD000258

[grl62242-bib-0013] Gristey, J. J. , Chiu, J. C. , Gurney, R. J. , Morcrette, C. J. , Hill, P. G. , Russell, J. E. , & Brindley, H. E. (2018). Insights into the diurnal cycle of global Earth outgoing radiation using a numerical weather prediction model. Atmospheric Chemistry and Physics, 18, 5129–5145. 10.5194/acp-18-5129-2018

[grl62242-bib-0014] Guevara, M. , Jorba, O. , Soret, A. , Petetin, H. , Bowdalo, D. , Serradell, K. , et al. (2021). Time‐resolved emission reductions for atmospheric chemistry modelling in Europe during the COVID‐19 lockdowns. Atmospheric Chemistry and Physics, 21, 773–797. 10.5194/acp-21-773-2021

[grl62242-bib-0015] Harries, J. E. , Russell, J. E. , Hanafin, J. A. , Brindley, H. , Futyan, J. , Rufus, J. , et al. (2005). The geostationary earth radiation budget project. Bulletin of the American Meteorological Society, 86, 945–960. 10.1175/BAMS-86-7-945

[grl62242-bib-0016] Haywood, J. M. , Allan, R. P. , Bornemann, J. , Forster, P. M. , Francis, P. N. , Milton, S. , et al. (2009). A case study of the radiative forcing of persistent contrails evolving into contrail‐induced cirrus. Journal of Geophysical Research, 114, D24201. 10.1029/2009JD012650

[grl62242-bib-0017] Heymsfield, A. , Winker, D. , Avery, M. , Vaughan, M. , Diskin, G. , Deng, M. , et al. (2014). Relationships between ice water content and volume extinction coefficient from in situ observations for temperatures from 0° to −86°C: Implications for spaceborne lidar retrievals. Journal of Applied Meteorology and Climatology, 53(2), 479–505. 10.1175/JAMC-D-13-087.1

[grl62242-bib-0018] Hogan, R. J. , & Bozzo, A. (2018). A flexible and efficient radiation scheme for the ECMWF model. Journal of Advances in Modeling Earth Systems, 10, 1990. 10.1029/2018MS001364

[grl62242-bib-0019] ICAO . (2021). Effects of novel coronavirus (COVID‐19) on civil aviation: Economic impact analysis (pp. 125.), Montreal. Retrieved from https://www.icao.int/sustainability/Documents/Covid-19/ICAO_coronavirus_Econ_Impact.pdf

[grl62242-bib-0020] Kärcher, B. (2018). Formation and radiative forcing of contrail cirrus. Nature Communications, 9, 1824. 10.1038/s41467-018-04068-0 PMC5940853 29739923

[grl62242-bib-0022] Lee, D. S. , Fahey, D. W. , Skowron, A. , Allen, M. R. , Burkhardt, U. , Chen, Q. , et al. (2021). The contribution of global aviation to anthropogenic climate forcing for 2000 to 2018. Atmospheric Environment, 244, 117834. 10.1016/j.atmosenv.2020.117834 32895604 PMC7468346

[grl62242-bib-0023] Lee, D. S. , Pitari, G. , Grewe, V. , Gierens, K. , Penner, J. E. , Petzold, A. , et al. (2010). Transport impacts on atmosphere and climate: Aviation. Atmospheric Environment, 44(37), 4678–4734. 10.1016/j.atmosenv.2009.06.005 32288556 PMC7110594

[grl62242-bib-0021] Le Quéré, C. , Jackson, R. B. , Jones, M. W. , Smith, A. J. P. , Abernethy, S. , Andrew, R. M. , et al. (2020). Temporary reduction in daily global CO_2_ emissions during the COVID‐19 forced confinement. Nature Climate Change, 10, 647–653. 10.1038/s41558-020-0797-x

[grl62242-bib-0024] Mannstein, H. , Brömser, A. , & Bugliaro, L. (2010). Ground‐based observations for the validation of contrails and cirrus detection in satellite imagery. Atmospheric Measurement Techniques, 3, 655–669. 10.5194/amt-3-655-2010

[grl62242-bib-0025] Markowicz, K. M. , & Witek, M. L. (2011). Simulations of contrail optical properties and radiative forcing for various crystal shapes. Journal of Applied Meteorology and Climatology, 50, 1740–1755. 10.1175/2011JAMC2618.1

[grl62242-bib-0026] Meerkötter, R. , Schumann, U. , Doelling, D. R. , Minnis, P. , Nakajima, T. , & Tsushima, Y. (1999). Radiative forcing by contrails. Annales Geophysicae, 17(8), 1080–1094. 10.1007/s00585-999-1080-7

[grl62242-bib-0027] Meyer, R. , Mannstein, H. , Meerkötter, R. , Schumann, U. , & Wendling, P. (2002). Regional radiative forcing by line‐shaped contrails derived from satellite data. Journal of Geophysical Research, 107(D10), 4104. 10.1029/2001JD000426

[grl62242-bib-0028] Minnis, P. , Ayers, J. K. , Palikonda, R. , & Phan, D. (2004). Contrails, cirrus trends, and climate. Journal of Climate, 17, 1671–1685. 10.1175/1520-0442(2004)017<1671:CCTAC>2.0.CO;2

[grl62242-bib-0029] Minnis, P. , Schumann, U. , Doelling, D. R. , Gierens, K. M. , & Fahey, D. W. (1999). Global distribution of contrail radiative forcing. Geophysical Research Letters, 26(13), 1853–1856. 10.1029/1999GL900358

[grl62242-bib-0030] Myhre, G. , Kvalevåg, M. , Rädel, G. , Cook, J. , Shine, K. P. , Clark, H. , et al. (2009). Intercomparison of radiative forcing calculations of stratospheric water vapour and contrails. Metz, 18(6), 585–596. 10.1127/0941-2948/2009/0411

[grl62242-bib-0031] Myhre, G. , & Stordal, F. (2001). On the tradeoff of the solar and thermal infrared radiative impact of contrails. Geophysical Research Letters, 28(6), 3119–3122. 10.1029/2001GL013193

[grl62242-bib-0032] Nuic, A. , Poles, D. , & Mouillet, V. (2010). BADA: An advanced aircraft performance model for present and future ATM systems. International Journal of Adaptive Control and Signal Processing, 24, 850–866. 10.1002/acs.1176

[grl62242-bib-0033] Penner, J. E. , Lister, D. H. , Griggs, D. J. , Dokken, D. J. , & McFarland, M. (1999). Aviation and the global atmosphere—A special report of IPCC working groups I and III. Intergovernmental panel on climate change (pp. 365). Cambridge, UK: Cambridge University Press. https://www.ipcc.ch/report/aviation-and-the-global-atmosphere-2/

[grl62242-bib-0034] Penner, J. E. , Zhou, C. , Garnier, A. , & Mitchell, D. L. (2018). Anthropogenic aerosol indirect effects in cirrus clouds. Journal of Geophysical Research: Atmospheres, 123, 11652–11677. 10.1029/2018JD029204 PMC6360521 30775191

[grl62242-bib-0035] Poll, D. I. A. , & Schumann, U. (2021). An estimation method for the fuel burn and other performance characteristics of civil transport aircraft during cruise: Part 2, determining the aircraft's characteristic parameters. Aeronautical Journal, 125(1284), 296–340. 10.1017/aer.2020.124

[grl62242-bib-0036] Sanz‐Morère, I. , Eastham, S. D. , Allroggen, F. , Speth, R. L. , & Barrett, S. R. H. (2021). Impacts of multi‐layer overlap on contrail radiative forcing. Atmospheric Chemistry and Physics, 21, 1649–1681. 10.5194/acp-21-1649-2021

[grl62242-bib-0037] Sausen, R. , Gierens, K. , Ponater, M. , & Schumann, U. (1998). A diagnostic study of the global distribution of contrails part I: Present day climate. Theoretical and Applied Climatology, 61, 127–141. 10.1007/s007040050058

[grl62242-bib-0038] Sausen, R. , Isaksen, I. , Grewe, V. , Hauglustaine, D. , Lee, D. S. , Myhre, G. , et al. (2005). Aviation radiative forcing in 2000: An update on IPCC (1999). Metz, 14, 555–561. 10.1127/0941-2948/2005/0049

[grl62242-bib-0039] Schmetz, J. , Pili, P. , Tjemkes, S. , Just, D. , Kerkmann, J. , Rota, S. , & Ratier, A. (2002). An introduction to Meteosat Second Generation (MSG). Bulletin of the American Meteorological Society, 83, 977–992. 10.1175/BAMS-83-7-Schmetz-210.1175/1520-0477(2002)083<0977:aitmsg>2.3.co;2

[grl62242-bib-0040] Schumann, U. (1996). On conditions for contrail formation from aircraft exhausts. Meteorologische Zeitschrift, 5(1), 4–23. 10.1127/metz/5/1996/4

[grl62242-bib-0041] Schumann, U. (2012). A contrail cirrus prediction model. Geoscientific Model Development, 5(3), 543–580. 10.5194/gmd-5-543-2012

[grl62242-bib-0042] Schumann, U. , & Graf, K. (2013). Aviation‐induced cirrus and radiation changes at diurnal timescales. Journal of Geophysical Research: Atmospheres, 118, 2404–2421. 10.1002/jgrd.50184

[grl62242-bib-0043] Schumann, U. , Graf, K. , & Mannstein, H. (2011). Potential to reduce the climate impact of aviation by flight level changes (pp. 1–22). AIAA paper 2011‐3376. 10.2514/6.2011-3376

[grl62242-bib-0044] Schumann, U. , & Heymsfield, A. J. (2017). On the life cycle of individual contrails and contrail cirrus. Meteorological Monographs, 58(3), 3.1–3.24. 10.1175/AMSMONOGRAPHS-D-16-0005.1

[grl62242-bib-0045] Schumann, U. , Mayer, B. , Graf, K. , & Mannstein, H. (2012). A parametric radiative forcing model for contrail cirrus. Journal of Applied Meteorology and Climatology, 51(6), 1391–1406. 10.1175/JAMC-D-11-0242.1

[grl62242-bib-0046] Schumann, U. , Penner, J. E. , Chen, Y. , Zhou, C. , & Graf, K. (2015). Dehydration effects from contrails in a coupled contrail‐climate model. Atmospheric Chemistry and Physics, 15, 11179–11199. 10.5194/acp-15-11179-2015

[grl62242-bib-0047] Schumann, U. , Poll, I. , Teoh, R. , Koelle, R. , Spinielli, E. , Molloy, J. , et al. (2021). Air traffic and contrail changes during COVID‐19 over Europe: A model study. Atmospheric Chemistry and Physics Discussions, 1–37. 10.5194/acp-2021-62

[grl62242-bib-0048] Spangenberg, D. A. , Minnis, P. , Bedka, S. T. , Palikonda, R. , Duda, D. P. , & Rose, F. G. (2013). Contrail radiative forcing over the Northern Hemisphere from 2006 Aqua MODIS data. Geophysical Research Letters, 40, 595–600. 10.1002/grl.50168

[grl62242-bib-0049] Stephens, G. L. (2005). Cloud feedbacks in the climate system: A critical review. Journal of Climate, 18(2), 237–273. 10.1175/JCLI-3243.1

[grl62242-bib-0050] Stordal, F. , Myhre, G. , Stordal, E. J. G. , Rossow, W. B. , Lee, D. S. , Arlander, D. W. , & Svendby, T. (2005). Is there a trend in cirrus cloud cover due to aircraft traffic? Atmospheric Chemistry and Physics, 5, 2155–2162. 10.5194/acp-5-2155-2005

[grl62242-bib-0051] Strandgren, J. , Bugliaro, L. , Sehnke, F. , & Schröder, L. (2017a). Cirrus cloud retrieval with MSG/SEVIRI using artificial neural networks. Atmospheric Measurement Techniques, 10, 3547–3573. 10.5194/amt-10-3547-2017

[grl62242-bib-0052] Strandgren, J. , Fricker, J. , & Bugliaro, L. (2017b). Characterisation of the artificial neural network CiPS for cirrus cloud remote sensing with MSG/SEVIRI. Atmospheric Measurement Techniques, 10, 4317–4339. 10.5194/amt-10-4317-2017

[grl62242-bib-0053] Stubenrauch, C. J. , & Schumann, U. (2005). Impact of air traffic on cirrus coverage. Geophysical Research Letters, 32, L14813. 10.1029/2005GL022707

[grl62242-bib-0054] Stuber, N. , Forster, P. , Rädel, G. , & Shine, K. (2006). The importance of the diurnal and annual cycle of air traffic for contrail radiative forcing. Nature, 441(7095), 864–867. 10.1038/nature04877 16778887

[grl62242-bib-0055] Teoh, R. , Schumann, U. , & Stettler, M. E. J. (2020). Beyond contrail avoidance: Efficacy of flight altitude changes to minimise contrail climate forcing. Aerospace, 7(9), 121. 10.3390/aerospace7090121

[grl62242-bib-0056] Teoh, R. , Stettler, M. E. J. , Majumdar, A. , Schumann, U. , Graves, B. , & Boies, A. M. (2019). A methodology to relate black carbon particle number and mass emissions. Journal of Aerosol Science, 132, 44–59. 10.1016/j.jaerosci.2019.03.006

[grl62242-bib-0057] Urbanek, B. , Groß, S. , Wirth, M. , Rolf, C. , Krämer, M. , & Voigt, C. (2018). High depolarization ratios of naturally occurring cirrus clouds near air traffic regions over Europe. Geophysical Research Letters, 45, 13166–13172. 10.1029/2018GL079345

[grl62242-bib-0058] van Heerwaarden, C. C. , Mol, W. B. , Veerman, M. A. , Benedict, I. , Heusinkveld, B. G. , Knap, W. H. , et al. (2021). Record high solar irradiance in Western Europe during first COVID‐19 lockdown largely due to unusual weather. Communications Earth and Environment, 2, 37. 10.1038/s43247-021-00110-0

[grl62242-bib-0059] Vázquez‐Navarro, M. , Mannstein, H. , & Kox, S. (2015). Contrail life cycle and properties from 1 year of MSG/SEVIRI rapid‐scan images. Atmospheric Chemistry and Physics, 15, 8739–8749. 10.5194/acp-15-8739-2015

[grl62242-bib-0060] Vázquez‐Navarro, M. , Mayer, B. , & Mannstein, H. (2013). A fast method for the retrieval of integrated longwave and shortwave top‐of‐atmosphere upwelling irradiances from MSG/SEVIRI (RRUMS). Atmospheric Measurement Techniques, 6, 2627–2640. 10.5194/amt-6-2627-2013

[grl62242-bib-0061] Voigt, C. , Lelieveld, J. , Schlager, H. , Curtius, J. , Bugliaro, L. , Erbertseder, T. , et al. (2020). BLUESKY—The aircraft mission on atmospheric composition changes in Europe during the Corona Lockdown 2020. In AGU Fall Meeting 1–17 December 2020. Retrieved from https://agu.confex.com/agu/fm20/webprogram/Paper728363.html. https://www.dlr.de/content/en/articles/news/2020/02/20200520_fewer-condensation-trails-due-to-reduced-air-traffic.html

[grl62242-bib-0062] Wielicki, B. A. , Barkstrom, B. R. , Harrison, E. F. , Lee, R. B. III , Louis Smith, G. , & Cooper, J. E. (1996). Clouds and the earth's radiant energy system (CERES): An earth observing system experiment. Bulletin of the American Meteorological Society, 77, 853–868. 10.1175/1520-0477(1996)077<0853:CATERE>2.0.CO;2

[grl62242-bib-0063] Winker, D. M. , Vaughan, M. A. , Omar, A. , Hu, Y. , Powell, K. A. , Liu, Z. , et al. (2009). Overview of the CALIPSO mission and CALIOP data processing algorithms. Journal of Atmospheric and Oceanic Technology, 26, 2310–2323. 10.1175/2009JTECHA1281.1

[grl62242-bib-0064] Zerefos, C. S. , Eleftheratos, K. , Balis, D. S. , Zanis, P. , Tselioudis, G. , & Meleti, C. (2003). Evidence of impact of aviation on cirrus cloud formation. Atmospheric Chemistry and Physics, 3, 1633–1644. 10.5194/acp-3-1633-2003

[grl62242-bib-0065] Zhou, C. , & Penner, J. E. (2014). Aircraft soot indirect effect on large‐scale cirrus clouds: Is the indirect forcing by aircraft soot positive or negative? Journal of Geophysical Research: Atmospheres, 119, 11303–11320. 10.1002/2014JD021914

