# Supplementary material for: Aviation Contrail Cirrus and Radiative Forcing Over Europe During 6 Months of COVID‐19
Source: Geophys Res Lett. 2021 Apr 28;48(8):e2021GL092771. doi: 10.1029/2021GL092771 (PMC8250229; doi:10.1029/2021GL092771)
Supplement: Supplementary file 1 — Supporting Information S1 [file GRL-48-e2021GL092771-s001.pdf]

AGU PUBLICATIONS

Geophysical Research Letters

Supplemental Information for

Aviation contrail cirrus and radiative forcing over Europe during six months of COVID-19

U. Schumann[1], L. Bugliaro[1], A. Dörnbrack[1], R. Baumann[1], and C. Voigt[1,2]

[1]Institute of Atmospheric Physics, Deutsches Zentrum für Luft- und Raumfahrt, 82234 Oberpaffenhofen, Germany.

[2]Johannes Gutenberg-University, Mainz, Germany.

Corresponding author: Ulrich Schumann (Ulrich.schumann@dlr.de)

Table of Contents

S1 Differences of interannual differences of observed and modelled fields 2
S2 Pearson's correlation r^2, normalized mean bias NMB, and root-mean-square error RMSE, definitions 3
S3 Weekly Cycle 4
S4 Air Traffic and Fuel density 5
S5 Mean values and mean differences of weather parameters in 2019 and 2020 from IFS data. 6
S6 Global Solar Surface Irradiance 8
S7 Potential Contrail Cover 9
S8 Acronyms 10
S9 Data Depository 11
References in this Supplement 12

S1 Differences of interannual differences of observed and modelled fields

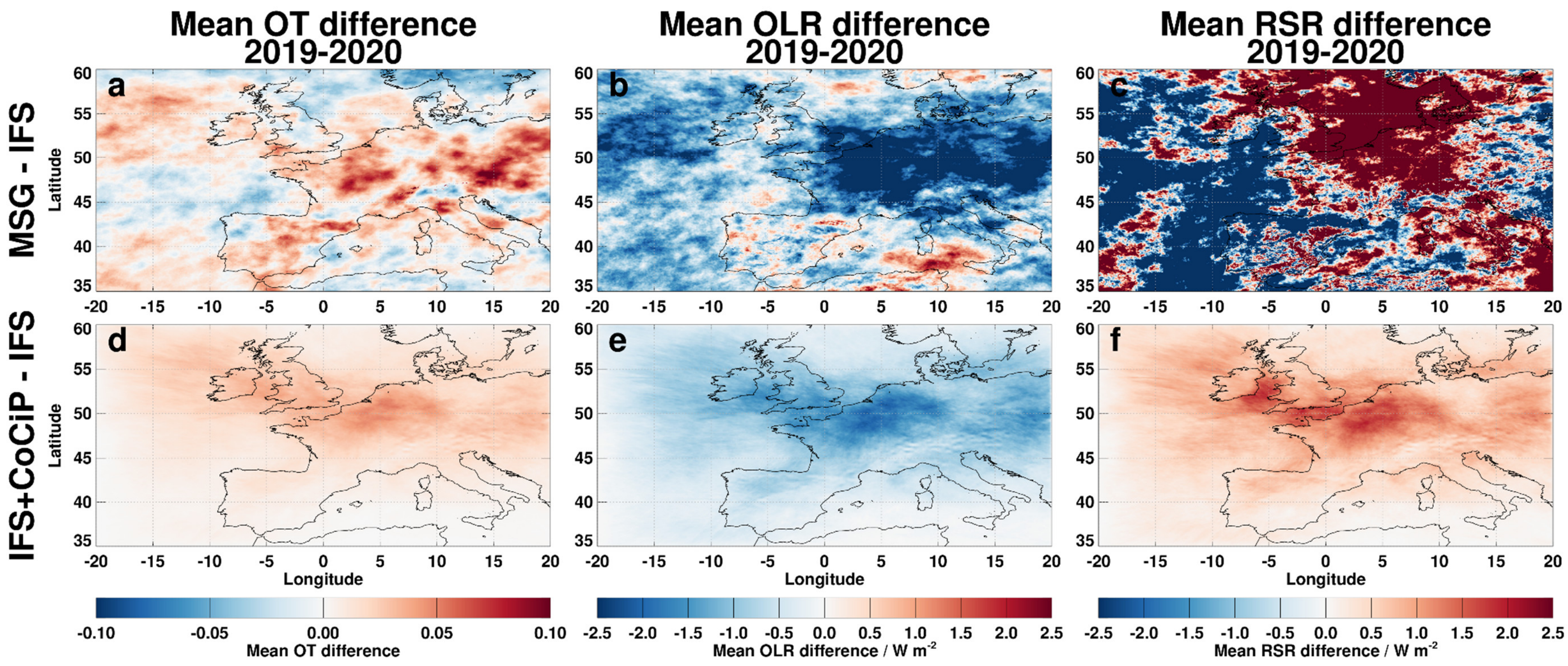

Figure S1. Mean differences between observation minus IFS (top) and (CoCiP+IFS) minus IFS (bottom), of March-August 2019-2020 differences of OT, OLR, and RSR. These data result from those in Figure 2, by taking the difference between the first and third row and the second and the third row. The min/max values in the panels a to f in the given units are as follows: -0.1/0.2; -5.2/3.3; -32.3/21.1; 0.0/0.054; -2.2/0.; 0./2.0. The colour scales resolve only subranges of these data ranges. The high observation variability is not covered by the models. The top row shows the difference between Meteosat-derived observations and IFS model results. The model results in the bottom row are now representing the pure CoCiP contrail results. The two rows now allow to identify signature similarities due to aviation contrails in the difference between Meteosat-derived observations and IFS model results. The observations suggest larger amplitudes of the aviation signature. This may well be the case, but it may be too early to conclude on this in view of the limited observation time period considered so far.

S2 Pearson's correlation r^2, normalized mean bias NMB, and root-mean-square error RMSE, definitions

For N given observations O_i (of arbitrary sign) and model predictions P_i, $i = 1,2,..,N$ (Emery et al., 2017):

$$r^2 = \frac{\sum(O_i - O_m)(P_i - P_m)}{\sqrt{\sum(O_i - O_m)^2}\sqrt{\sum(P_i - P_m)^2}}, \text{ with } P_m = \frac{\sum P_i}{N}, O_m = \frac{\sum O_i}{N}$$

$$\text{NMB} = \frac{\frac{1}{N}\sum(P_i - O_i)}{\sqrt{\frac{\sum {O_i}^2}{N}}}$$

$$\text{RMSE} = \sqrt{\frac{\sum(P_i - O_i)^2}{N}}$$

Different from Emery et al. (2017), the NMB is related to the root-mean-square value of the observations, because in the present application the observations can be both positive and negative.

S3 Weekly Cycle

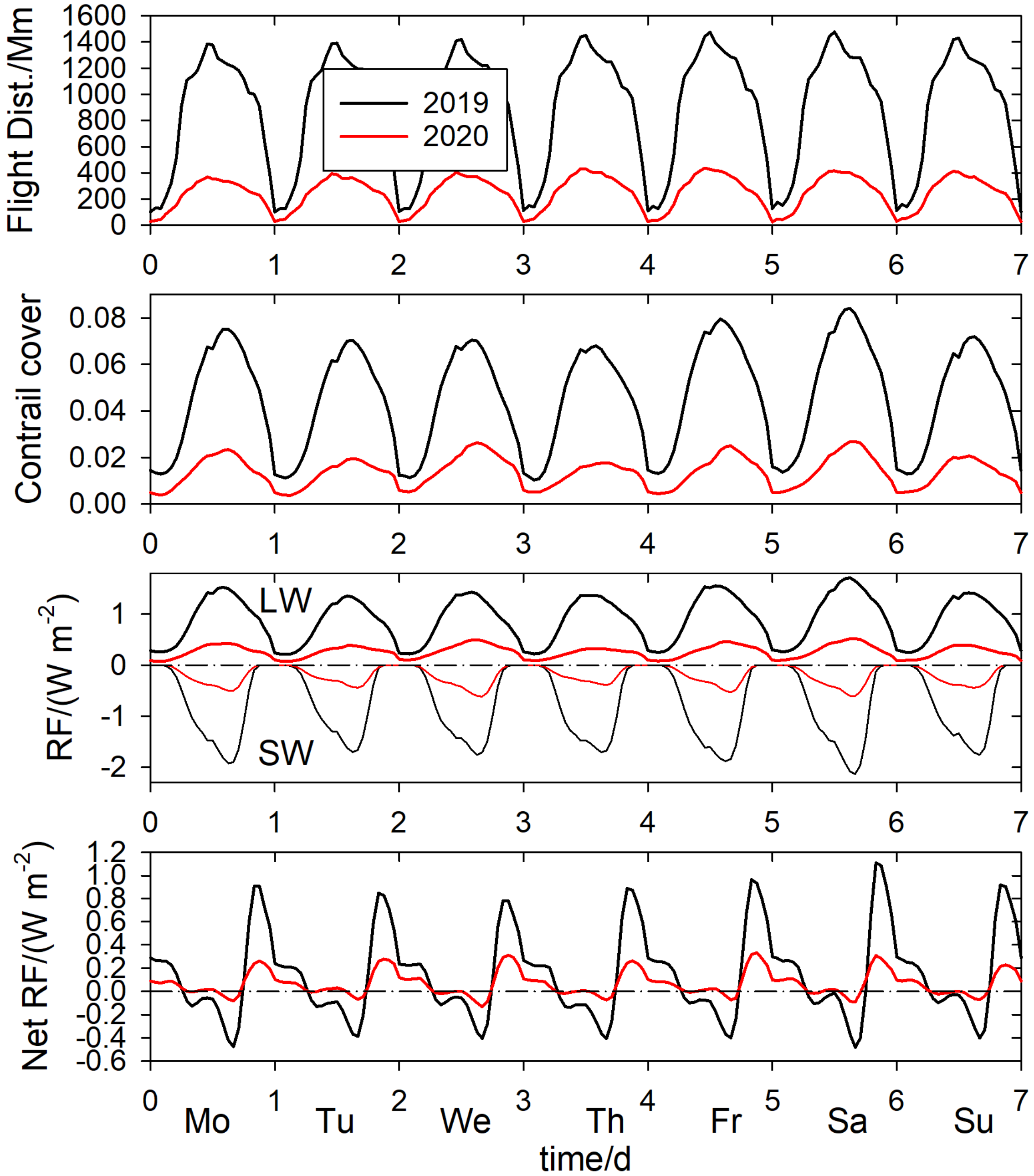

Figure S3. Weekly traffic and contrail cycles with hourly resolution from 26 weeks between March and August in 2019 and 2020. Mean values in the European investigation domain are plotted versus time for weekdays from Monday to Sunday (top to bottom) for flight distance above FL 180, cover of contrail cirrus with optical thickness > 0.1, longwave (LW), shortwave (SW), and net (SW+LW) RF.

S4 Air Traffic and Fuel density

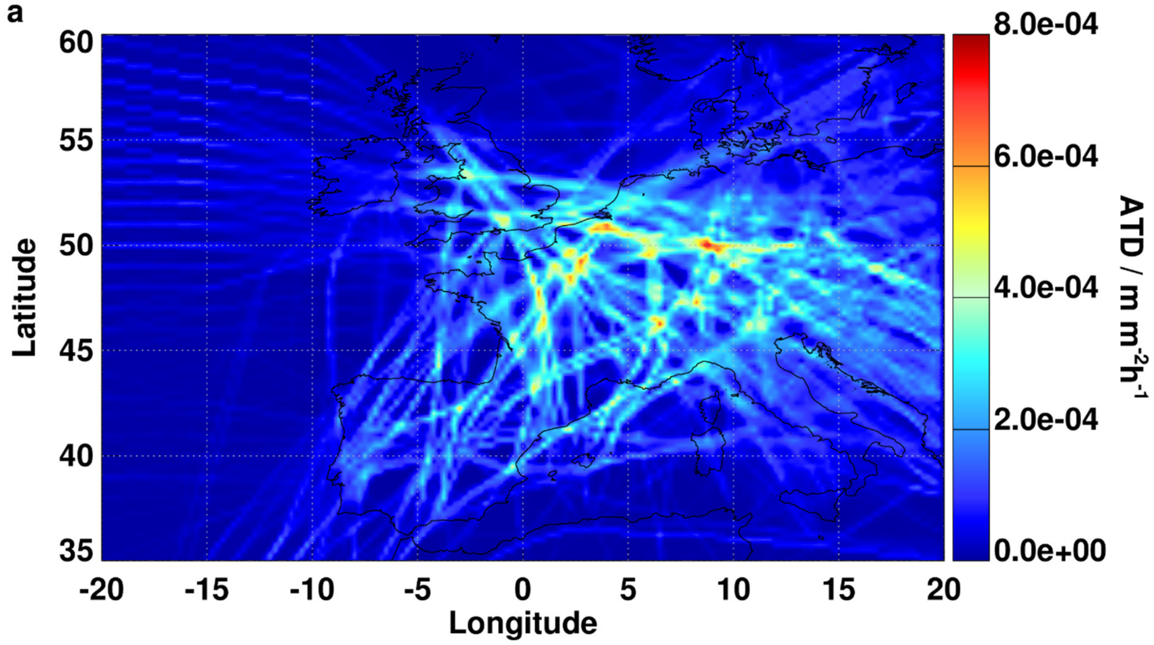

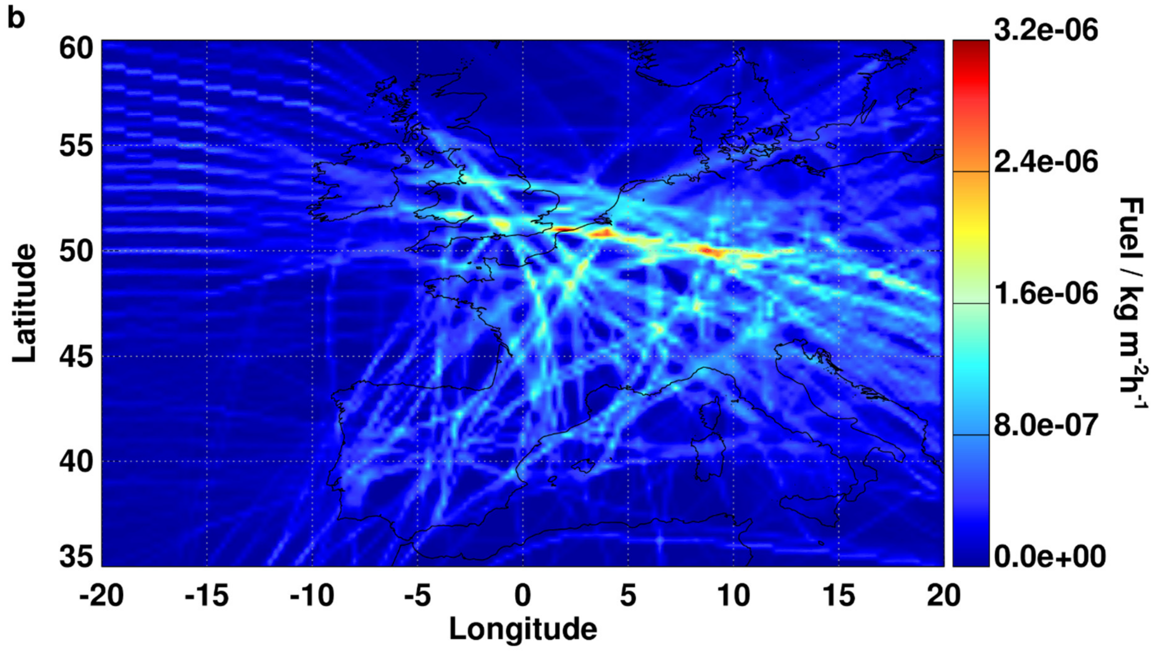

Figure S4. Mean differences of air traffic density (a) and fuel consumption (b) above FL 180 over Europe between March-August 2019 and March-August 2020.

S5 Mean values and mean differences of weather parameters in 2019 and 2020 from IFS data.

Table S5 provides mean values at TOA: Outgoing Longwave Radiation, Reflected Solar Radiation, and planetary albedo (= RSR/SDR, SDR = incoming solar radiation); at Surface: pressure, temperature, and global solar irradiance; and at Flight Level 350 (10.7 km, 238 hPa): geopotential height, temperature, absolute humidity, potential contrail cover (i.e., fractional area in which the temperature is below the threshold temperature for contrail formation and the humidity exceeds ice saturation), and upward vertical wind. The mean values are taken over two inclined parallel subdomains (see Figure S5), each of 7.5° width in North-South direction above (N) or below (S) about 47.5°N. The central domain boundary is the line between 20°W, 52.5°N and 20°E, 42.5°N. The mean and difference values (mean, diff) refer to different seasons in the years 2019 and in the 2020-2019 difference.

Table S5. Mean 2019 values and 2020-2019 differences in meteorological parameters in March-May and June-August, north (N) or south (S) of about 47.5°N.

Season		MAM				JJA			
Domain		N		S		N		S	
Parameter	Unit	mean	diff	mean	diff	mean	diff	mean	diff
TOA:									
OLR	W m^{-2}	229.8	8.3	237.0	2.0	234.2	2.8	249.3	1.2
RSR	W m^{-2}	113.4	-14.7	107.6	-0.2	131.5	-2.0	106.9	1.9
Albedo	%	27.0%	-2.5%	25.3%	-0.2%	29.7%	-0.5%	25.9%	0.2%
Surface									
Pressure	hPa	994.2	2.7	998.1	0.4	992.6	1.0	997.7	0.2
Temperature	K	282.5	0.3	286.2	0.8	285.8	-0.2	289.5	0.2
Solar Irradiance	W m^{-2}	185.89	21.0	207.2	0.4	211.4	-14.6	262.6	-6.5
At FL 350									
Geopotential	m	10656	67	10767	52	10777	32	10912	31
Temperature	K	218.2	0.8	218.9	1.0	220.3	0.2	221.5	0.7
Humidity	µg g^{-1}	22.17	0.91	26.56	1.29	35.93	1.13	39.22	-0.04
Potential Contrail Cover	%	15.1%	-1.2%	14.9%	-1.2%	16.7%	-0.2%	12.9%	-0.7%
Uplifting Wind	m s^{-1}	-0.51	-1.29	0.07	0.71	-0.05	-0.54	-1.79	-0.21

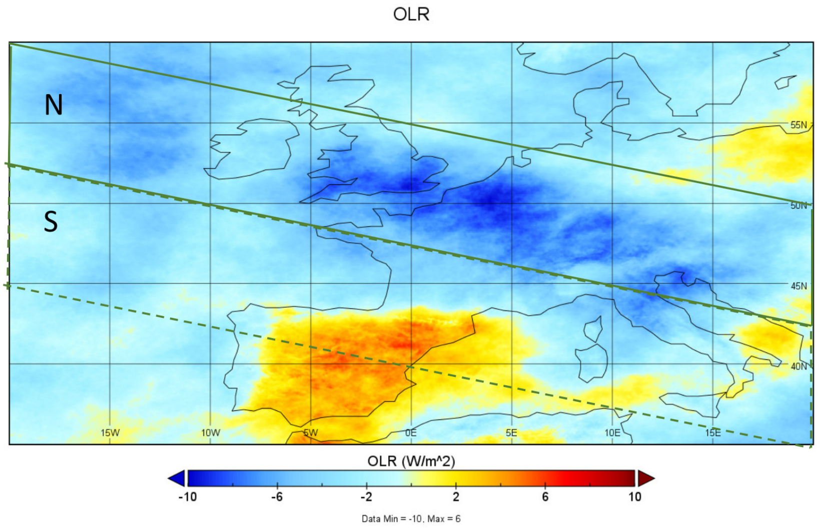

Figure S5. N-S-Domain definition referred to in Table S5. Northern and Southern domains (N, S) referred to in the table, here overlaid on the mean OLR difference in March-August between 2019 and 2020.

S6 Global Solar Surface Irradiance

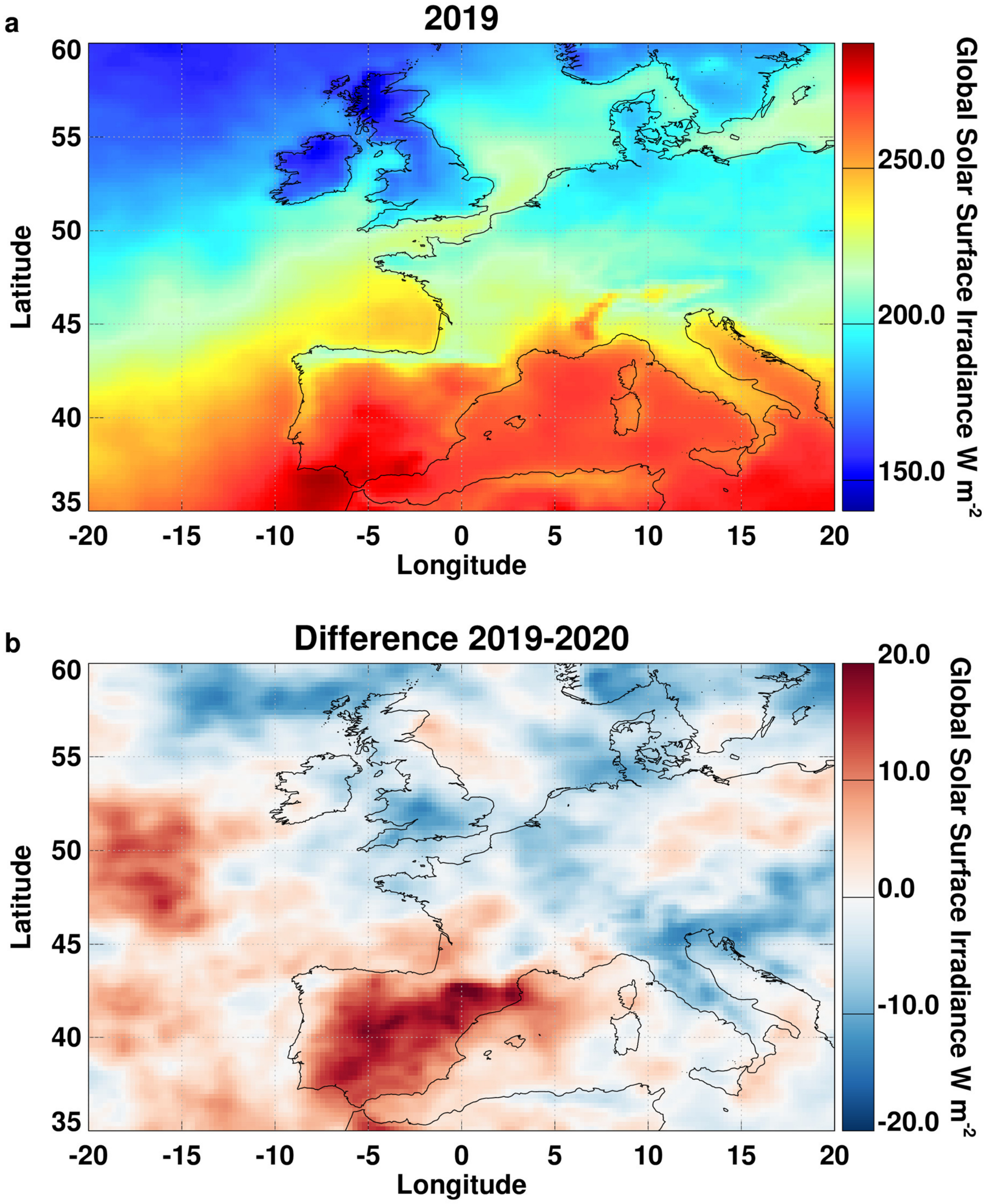

Figure S6. Global solar irradiance at the surface on average over March-August in 2019 (top, 143 to 290 W m^{-2}) and in the 2019-2020 difference (bottom, -14.6 to 21 W m^{-2}). The blue area in the bottom figure marks the "blue-sky" area.

S7 Potential Contrail Cover

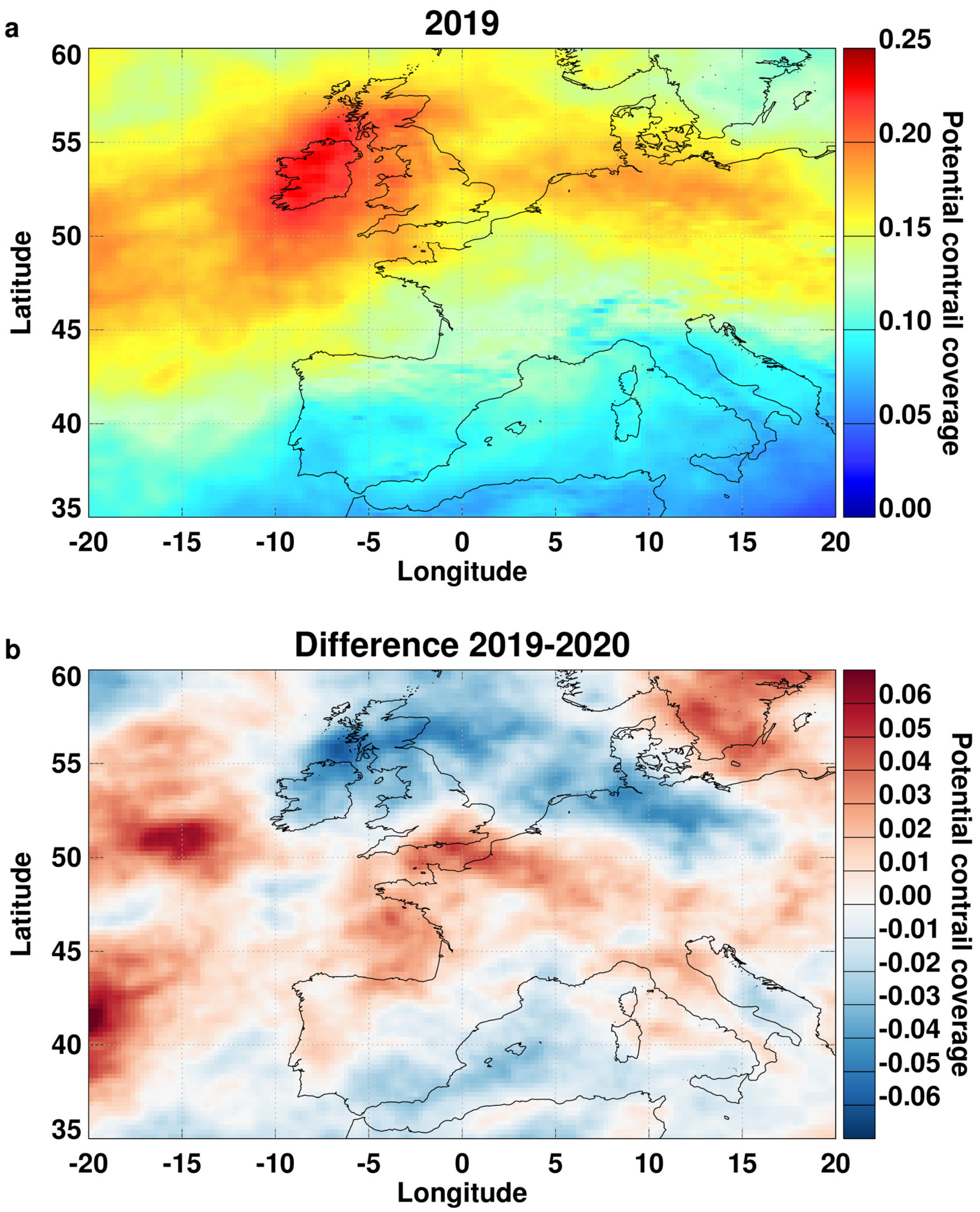

Figure S7. Potential contrail coverage (up to 23%) at FL 350 for March-August in 2019 (top) and in the 2019-2020 difference (-6.1 to 7.3%, bottom).

S8 Acronyms

Acronym	Explanation
BADA3	Base of Aircraft Data version 3
CERES	Clouds and the Earth's Radiant Energy System
CiPS	Cirrus Properties from SEVIRI
CoCiP	Contrail Cirrus Prediction Model
ECMWF	European Centre for Medium-Range Weather Forecasts
EUROCONTROL	European Organization for the Safety of Air Navigation
FL	Flight Level
GERB	Geostationary Earth Radiation Budget
ICAO	International Civil Aviation Organization
IFS	Integrated Forecasting System
LW	Longwave
METEOSAT	Meteorological Satellite
MSG	METEOSAT Second-Generation
NATS	Navigation service provider
OLR	Outgoing Longwave Radiation
OT	Optical Thickness
PS	Performance Model
RF	Radiative Forcing
rms	root mean square
RRUMS	Rapid Retrieval of upwelling irradiances from MSG-SEVIRI
RSR	Reflected Shortwave Radiation
SDR	Solar Direct Radiation
SEVIRI	Spinning Enhanced Visible and Infrared Radiometer
SW	Shortwave
SZA	Solar Zenith Angle
TOA	Top of Atmosphere
UK	United Kingdom

S9 Data Depository

The data suppository contains netcdf files with 6-month mean values from MSG-SEVIRI observations and from the CoCiP/IFS model simulations for the reference case (Schumann, 2021). The zipped file has a size of 46 MB.

The files
MSG2019.nc
MSG2020.nc
contain the
MSG SEVIRI data:

6-month mean values for March-August 2019 and 2020 versus latitude and longitude,
for
IOT - ice cloud 550 nm optical thickness (nondimensional)
OLR - outgoing longwave radiation (W m^{-2})
RSR - reflected solar radiation (W m^{-2})
with related latitude and longitude coordinates.

The files
COCIPC95X19CP2019.nc
COCIPC95X20CP2020.nc
contain the
6-month mean values of CoCiP model output versus latitude and longitude for the reference case as described in Schumann et al. (2021),
for
ATD - Air traffic density in m m^{-2} h^{-1}
FUEL - fuel consumption in kg m^{-2} h^{-1}
PC - potential contrail coverage thickness in m.
TAUCO - optical depth of contrails
TAUCI - optical depth of cirrus
TAUCICO - optical depth of sum of cirrus and contrails from IFS and CoCiP
RFLW - Longwave Radiative Forcing in W m^{-2}
RFSW - Shortwave Radiative Forcing in W m^{-2}
RFnet - Net Radiative Forcing in W m^{-2}
OLRCI - outgoing longwave radiation from IFS (cirrus)
RSRCI - reflected solar radiation from IFS (cirrus)
OLRCICO - outgoing longwave radiation from IFS and CoCiP (sum of cirrus and contrails)
RSRCICO - reflected solar radiation from IFS and CoCiP (sum of cirrus and contrails)

References in this Supplement

Emery, C., Z. Liu, A. G. Russell, M. T. Odman, G. Yarwood, and N. Kumar (2017). Recommendations on statistics and benchmarks to assess photochemical model performance. *Journal of the Air & Waste Management Association*, *67*(5), 582-598, doi: 10.1080/10962247.2016.1265027.

Schumann, U. (2021). Aviation contrail cirrus and radiative forcing over Europe for six months in 2020 during COVID-19 compared with 2019: Observations and model results (Version1) [Data set]. Zenodo. http://doi.org/10.5281/zenodo.4481680, edited, doi: 10.5281/zenodo.4481680.

Schumann, U., I. Poll, R. Teoh, R. Koelle, E. Spinielli, J. Molloy, G. S. Koudis, R. Baumann, L. Bugliaro, M. Stettler, and C. Voigt (2021). Air traffic and contrail changes during COVID-19 over Europe: A model study. *Atmos. Chem. Phys. Discuss.*, *https://doi.org/10.5194/acp-2021-62*, 1-37.
